# Enhanced arabinose utilization by adaptively evolved *Klebsiella pneumoniae* enables efficient 2,3-butanediol production from sugar beet pulp

**DOI:** 10.1039/d6se00474a

**Published:** 2026-07-12

**Authors:** Kawinharsun Dhodduraj, Vivek Narisetty, Seyed Ali Nabavi, Frederic Coulon, Sunil K. Maity, Vinod Kumar

**Affiliations:** a Faculty of Engineering and Applied Sciences, Cranfield University Cranfield MK43 0AL UK Vinod.Kumar@cranfield.ac.uk s.nabavi@cranfield.ac.uk +44 (0) 1234754786 +44 (0) 1234754225; b Department of Chemical Engineering, Indian Institute of Technology Hyderabad Kandi Sangareddy-502284 Telangana India; c Centre for Sustainable Rural Development, Indian Institute of Technology Roorkee Roorkee Uttarakhand India

## Abstract

Fermentative production of 2,3-butanediol (BDO) offers a sustainable alternative to fossil-based chemical synthesis, but its economic feasibility strongly depends on the use of low-cost feedstocks and efficient microbial strains capable of utilizing mixed sugars. In the current study, *Klebsiella pneumoniae* was used to produce BDO from arabinose and glucose-rich fractions derived from sugar beet pulp (SBP), a major sugar industry waste. Initial shake-flask studies revealed efficient BDO formation from arabinose, although fermentation performance was declined at higher concentrations (60 g L^−1^) due to substrate inhibition. The strain was subjected to adaptive laboratory evolution (ALE) to overcome this limitation. The evolved strain (A4) showed enhanced arabinose utilization and achieved a productivity of 0.54 g L^−1^ h^−1^ at 100 g L^−1^ arabinose compared with 0.43 g L^−1^ h^−1^ for the parent strain. The fed-batch bioreactor cultivation of the evolved strain resulted in BDO titers of 84.9 and 96.7 g L^−1^ with a conversion yield of 0.39 g g^−1^ using arabinose- and glucose-rich mixture of pure sugars, respectively. During fed-batch cultivation, the strain accumulated 82.2 and 92.4 g L^−1^ BDO from arabinose- and glucose-rich SBP hydrolysates, respectively, with a conversion yield of 0.38 g g^−1^. The pasteurization of SBP hydrolysates significantly improved fermentation kinetics, which resulted in the shortening of fermentation times by 30–40 h while maintaining the BDO titer and yield comparable to the non-pasteurized hydrolysate. Overall, BDO production from pure sugars and sugar-rich hydrolysates from SBP was similar. These results demonstrate the immense potential of the isolate *K. pneumoniae* as a biofactory in valorizing SBP into BDO with possibility for future industrialization.

## Abstract 2

This study underscores the immense potential of *Klebsiella pneumoniae* as a robust cell factory for efficient valorization of arabinose- and glucose-rich sugar beet pulp hydrolysates into 2,3-butanediol, a product with huge commercial potential.

## Graphical abstract

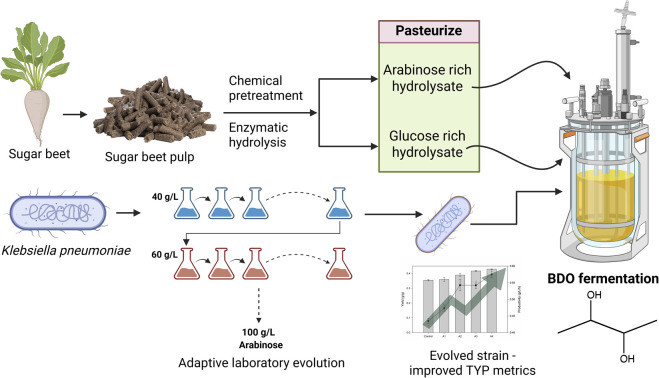

## 1. Introduction

In the present time, we are overdependent on fossil fuels, and their massive use is associated with a plethora of environmental and societal problems as well as rapid depletion of finite fossil resources. One of the main issues with the fossil-based production of fuels and chemicals is global warming caused by the significant release of carbon emissions into the environment.^1,2^ This has created a push on industries and society to make a transition from a fossil-based economy towards bioeconomy with the smart use of available resources. To envisage a society free of fossil carbon, it is essential to utilize renewable carbon sources to decarbonize our production routes, ultimately aiming for a carbon-neutral society.^3,4^ The concept of biorefineries gives the opportunity to reconfigure synthetic routes with greener alternatives using renewable feedstocks in pure as well as crude forms. Biomass, a product of CO_2_ fixation, is highly abundant and stands out as a renewable source with the potential to serve as a feedstock for sustainable biorefineries to achieve net zero production of chemicals.^5^ This is the era of circular economy, which is based on closed loop flow of materials to improve resource efficiency for extracting their maximum value, minimizing the volume of waste and simultaneously protecting primary raw materials while decoupling economic growth from the negative consequences of poor resource management. Circular economy, creating wealth from waste, is an alternative to unsustainable linear economy and requires renewable resources. The decarbonisation of the chemical sector with a circular economy approach is an interesting proposition, the benefits of which are copious.^6–8^ A significant amount of CO_2_ is absorbed during the formation of lignocellulosic biomass (LCB), the most abundant material on the Earth. Therefore, the conversion of LCB to chemical building blocks can help in addressing the carbon emission problem.^9^

Sugar beet is a major agricultural crop containing approximately 20% w/w sucrose in its roots. It is cultivated in several parts of the world, including Europe and North America, to produce sugar. Sugar beet pulp (SBP) is a fibrous, sugar-depleted material discarded after the extraction of sugar from sliced beetroots and major waste stream from the sugar industries using sugar beet.^10^ Being a rich and renewable source of polysaccharides with a low lignin content, SBP has great biotechnological potential.^11^ SBP is an underutilized sustainable resource containing 65–80% polysaccharides (cellulose, hemicellulose, and pectin).^10^ The carbohydrate composition is as follows: 20–25% cellulose; 25–36% hemicellulose containing mainly arabinose with a small amount of xylose, mannose and galactose; and 18–30% pectin containing predominantly galacturonic acid with a small amount of arabinose and rhamnose. SBP is a low-lignin feedstock (<7%) with a considerable amount of protein (∼10%) and a small amount of fat and ash.^8,12^ The global sugar beet production was around 290 million tonnes in 2023. European Union is the leading producer of sugar beet with annual production of more than 110 million tonnes, and generates around 7 million tonnes of dried SBP.^10^ The production of one tonne of sugar requires processing of ∼6.6 tonnes of sugar beet. This generates around 500 kg of wet and 51–70 kg of dried pulp for every one tonne of sugar beet processed. The current applications of SBP include selling as low-cost feed for animals and beet pulp pellets to ensure long-term storage without spoiling, which entails high dehydrating and pelletizing cost.^8,13–15^

2,3-Butanediol (BDO) is a representative C4 chemical with two hydroxyl groups attached on second and third carbon atoms. It has a growing commercial market with applications in chemical, food and pharmaceutical industries. The market value was reported at USD 275 million in 2023 and is forecasted to reach more than USD 350 million by 2030.^16^ Due to the presence of two –OH groups, BDO is a versatile molecule as it can be transformed into numerous chemicals such as methyl ethyl ketone, isobutyraldehyde, 1,3-butadiene, and diacetyl. BDO has a heating value of 27.2 kJ g^−1^, therefore, BDO or some of its derivatives can be used as fuel derivatives and precursors for manufacturing biofuels including sustainable aviation fuels. The microbiological route using renewable feedstocks is a sustainable method for producing BDO.^17,18^ A limited number of companies such as LanzaTech (https://www.lanzatech.com) and GS Caltex Corporation (https://www.gscaltex.com) have started producing bio-based BDO on a large scale where BDO is manufactured using carbon monoxide from steelmaking and edible feedstocks (cassava and sugarcane), respectively. Even today, the biotechnology related to BDO production is concentrated at the laboratory level, and in order to implement the economically viable bioprocesses on a large scale, the manufacturing price of BDO should be ∼USD 1.0 per kg. Therefore, there is need for the development of BDO hyper producers cultivable on low-cost substrates.^2,19^

LCB is the most abundant material on the Earth and most research on its valorization *via* the microbial route focuses on the cellulosic part as many cell factories lack the ability to metabolize pentose sugars and other carbon sources found in LCB.^20^ For a profitable LCB-based biorefineries, it is important to valorize all the fractions of LCB. In the present study, BDO was manufactured using sugars obtained from hydrolysis of the cellulosic and hemicellulosic fractions of SBP by an environmental isolate of *Klebsiella pneumoniae*. *K. pneumoniae* is a versatile bioproduction platform for BDO synthesis from a variety of carbon sources including hexose and pentose sugars and disaccharides. In contrast, there are very few reports on using arabinose as a carbon source for the production of BDO under fermentative route.^10,20,21^ The work started with understanding the fermentation performance of *K. pneumoniae* using different concentrations of arabinose as the sole carbon source. Further, it was identified that at an arabinose concentration above 50 g L^−1^, there was significant substrate inhibition, causing reduced BDO yield and productivity. Therefore, the strain was subjected to adaptive laboratory evolution (ALE) to improve tolerance to high levels of arabinose and BDO production metrics from arabinose. The evolved strain was cultured at different concentrations of arabinose and glucose. This was followed by the pretreatment and depolymerisation of SBP to obtain arabinose and glucose-rich hydrolysates. These hydrolysates with different sugar levels were used for BDO production. After shake flask cultivation, the data were scaled up and fed-batch cultures were performed in bioreactors using pure arabinose-rich, glucose-rich sugar mixtures and SBP-derived sugar-rich hydrolysates.

## 2. Materials and methods

### 2.1 Materials

All the chemicals and media components used in this study were purchased from Sigma-Aldrich (Merck Life Science UK Limited) and are of analytical grade. The two distinct non-detoxified SBP hydrolysates generated in a separate study were used as fermentation substrates.^22^ The composition of the concentrated hydrolysates was as follows: the arabinose-rich hydrolysate contained 302.5 g L^−1^ arabinose, 23.8 g L^−1^ xylose, and 77.2 g L^−1^ glucose; the glucose-rich hydrolysate contained 40.3 g L^−1^ arabinose, 49.1 g L^−1^ xylose, and 302.6 g L^−1^ glucose. Additionally, two mixtures of pure sugar solutions (arabinose-rich and glucose-rich) were prepared to mimic the concentrations found in these hydrolysates for comparative studies.

### 2.2 Microorganisms, media, and culture conditions

The strain used in the current study was isolated at the Laboratory of Food Microbiology and Biotechnology at the Agricultural University of Athens, Greece and identified as *Klebsiella pneumoniae* by hybrid genome sequencing. The strain was maintained in a tryptic soy broth (TSB) with 30% (v/v) glycerol at −80 °C. The YPA medium used in this study consisted of 10 g L^−1^ yeast extract, 20 g L^−1^ peptone, and arabinose ranging from 40 to 100 g L^−1^. The concentration of arabinose was adjusted as required within this range. The BDO fermentation medium contained the following components (g L^−1^): (NH_4_)_2_HPO_4_, 6; (NH_4_)_2_SO_4_, 7.2; yeast extract, 2; KOH, 0.45; EDTA, 0.51; MgSO_4_·7H_2_O, 0.3; and (mg L^−1^): CaCl_2_·6H_2_O, 90; FeSO_4_·7H_2_O, 25; MnSO_4_·H_2_O, 3.8; ZnSO_4_·7H_2_O, 7.5.^20^ The sugar concentrations (glucose, xylose, and arabinose) were adjusted as per the experimental requirement. Unless otherwise stated, all fermentation experiments were performed under the following conditions: the initial pH was adjusted to 6.6 using 5 M NaOH prior to sterilization, the temperature was maintained at 30 °C, and agitation was set at 200 rpm. During fermentation, the pH was periodically readjusted to 6.6 whenever it dropped to ∼5.5.^23^ All the shake flask experiments were carried out in a 500 mL Erlenmeyer flask containing 100 mL fermentation media. The seed cultures for both shake flask and bioreactor experiments were prepared by inoculating a freshly sub-cultured colony of *K. pneumoniae* into TSB and incubating for 16 h under the conditions mentioned above.

### 2.3 Pretreatment and saccharification of SBP

SBP was subjected to hydrochloric acid hydrolysis for the extraction of arabinose-rich hydrolysates, while glucose-rich hydrolysates were produced by pre-treating SBP with a combination of hydrogen peroxide and Tween-80 followed by cellulase hydrolysis. The pretreatment conditions were previously optimized.^22^ Following the separation of sugar-rich liquid hydrolysates, both the hydrolysates were concentrated using rotary vacuum evaporation at 100 mbar and 60 °C and subsequently diluted according to the experimental requirements. When necessary, the hydrolysates were pasteurized at 70 °C ± 2 °C for 30 min. No sugar degradation products, such as furfural or 5-hydroxymethylfurfural (HMF), were detected in both hydrolysates.

### 2.4 Shake flask cultivation of *K. pneumoniae* on pure arabinose

The strain was cultivated at different concentrations of pure arabinose (10–60 g L^−1^) to evaluate the fermentative performance of *K. pneumoniae* and determine the effect of arabinose concentration on cell growth, substrate utilization, and metabolite production. Fermentation was carried out in shake flasks, as described in Section 2.2.

### 2.5 Adaptive laboratory evolution of *K. pneumoniae* and screening for BDO production

The objective of the ALE is to address the substrate inhibition caused by high concentrations of arabinose. To enhance the substrate tolerance, the strain was cultured in a YPA liquid medium (as described in Section 2.2) using four different concentrations of arabinose: 40, 60, 80, and 100 g L^−1^. The *K. pneumoniae* strain was initially cultivated with 40 g L^−1^ of arabinose and then propagated by gradually increasing the arabinose concentration in steps of 20 g L^−1^, until reaching 100 g L^−1^. Each step involved 20 days of subculturing every 24 hours in a fresh medium with the corresponding arabinose concentration, resulting in 20 passages per adaptation stage and a total of 80 passages throughout the ALE process. Briefly, the experiment began with the strain being grown in a YPA medium containing 40 g L^−1^ of arabinose, subculturing every 24 hours for 20 days. After this period, the resulting strain was transferred to a medium containing 60 g L^−1^ of arabinose, continuing the process similarly. Glycerol stocks of resulting strains were prepared using 20% (v/v) glycerol after each step and stored at −80 °C until further use. After each 20-day adaptation period, the resulting strain was screened for its ability to grow and produce BDO at higher arabinose concentrations. A 2% (v/v) seed culture of each strain was transferred into a 100 mL fermentation media (as described in Section 2.2) containing 100 g L^−1^ of arabinose, and the fermentation was conducted for around 80 h. The strain selection was performed based on the following key parameters: arabinose consumption, BDO titer, yield and productivity. The evolved strains obtained from 40, 60, 80 and 100 g L^−1^ arabinose adaptation stages were designated as A1, A2, A3 and A4, respectively.

### 2.6 Shake flask studies of evolved *K. pneumoniae* on a pure sugar mixture and SBP hydrolysates

The best performing strain was selected from the screening experiment for all the subsequent studies. The fermentation was carried out in the synthetic medium (as described in Section 2.2) supplemented with SBP hydrolysates and the mixtures of pure sugar (as described in Section 2.1). Both the hydrolysates and pure sugar mixtures were diluted to achieve a total sugar concentration of ∼80 g L^−1^.

### 2.7 Bioreactor studies of evolved *K. pneumoniae* on a pure sugar mixture and SBP hydrolysates

Fed-batch experiments were performed in a 2.5 L benchtop stirred tank bioreactor (Electrolab Bioreactors, UK) with a working volume of 1 L and an inoculum size of 10% (v/v). The aeration rate, agitation speed, and temperature were controlled at 1 vvm, 200 rpm, and 30 °C, respectively. The pH of the system was controlled in a cyclic manner (as described in Section 2.2). Fed-batch experiments were conducted using arabinose-rich and glucose-rich SBP hydrolysates. Two sets of hydrolysate fermentations were performed using pasteurized and non-pasteurized SBP hydrolysates to evaluate the effect of pasteurization on fermentation performance. In addition, fermentations using the corresponding pure sugar mixtures were conducted for comparison. The initial total sugar concentration in all experiments was ∼80 g L^−1^. In the fed-batch experiments, a constant volume of 1 L was maintained by removing an adequate amount of fermentation broth before each feeding. This allowed for intermittent substrate feeding to keep the residual sugar concentration above 10 g L^−1^. The feeding was carried out using a concentrated stock solution with a total sugar concentration of ∼400 g L^−1^.

### 2.8 Analytical methods

The samples were withdrawn periodically during the fermentation and centrifuged at 10 000 rpm for 10 min. The resulting cell pellet was resuspended in deionized water for cell growth analysis *via* optical density measurements at 600 nm. The supernatant was used for further analysis of residual sugars and metabolites using high-performance liquid chromatography (HPLC) (Agilent Technologies 1200 series, USA). A Rezex ROA-Organic Acid H^+^ column (Phenomenex, USA) was maintained at 60 °C and connected to a refractive index detector (RID) for analyzing sugars, BDO, acetoin, and ethanol, while a diode array detector (DAD) was used for organic acids (acetic, succinic, citric, formic, and lactic acid) analysis. The mobile phase consisted of 5.0 mM H_2_SO_4_, with flow rates of 0.4 mL min^−1^ for RID and 0.6 mL min^−1^ for DAD. Unless otherwise stated, the BDO yield is expressed as g BDO per g sugar consumed (g g^−1^).

### 2.9 Statistical significance

All the shake flask experiments were performed in triplicates, while the bioreactor runs were carried out in duplicates, with the standard deviation (SD) less than 10%. A one-way ANOVA was conducted to compare the results across different experimental groups, assessing the presence of statistically significant differences among group means. Pairwise comparisons were then conducted using Tukey's honest significant difference (HSD) test for *post-hoc* analysis to identify specific groups with significant differences. Statistical significance was determined at an alpha level of 0.05.

## 3. Results

### 3.1 Shake flask cultivation of *K. pneumoniae* on pure arabinose

**Fig. 1:**
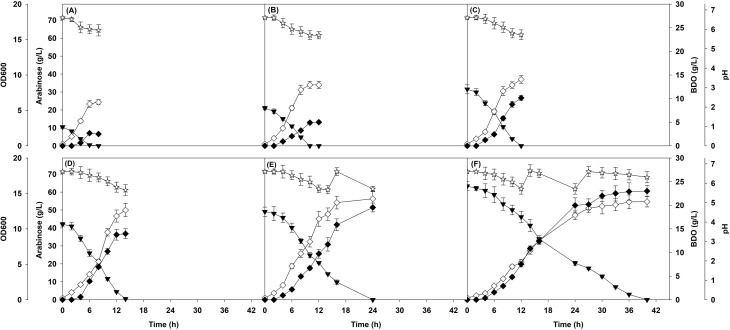
Shake flask cultivation of parent *K. pneumoniae* strain at different arabinose levels: (A) 10 g L^−1^; (B) 20 g L^−1^; (C) 30 g L^−1^; (D) 40 g L^−1^; (E) 50 g L^−1^; and (F) 60 g L^−1^. Symbols: arabinose (▼), OD_600_ (◇), BDO (◆), and pH (☆).

BDO is a well-known microbial fermentation product that can be synthesized by several microorganisms, including species of *Enterobacter*, *Bacillus*, and *Serratia*, among others. Among these, *Klebsiella pneumoniae* is one of the most extensively studied and efficient natural producers of BDO. This organism is metabolically versatile and capable of utilizing a wide range of carbon sources such as glucose, fructose, xylose, arabinose, glycerol, and sucrose. However, most BDO studies with *K. pneumoniae* focus on glucose or glycerol as the substrate. In contrast, reports describing BDO production from arabinose by *K. pneumoniae* remain limited.^5,21^ Therefore, BDO production from arabinose was evaluated in shake-flask cultures with different initial arabinose concentrations (10–60 g L^−1^). The time-course profiles for arabinose consumption, cell growth, BDO formation, and pH are presented in Fig. 1. Increasing the initial arabinose concentration resulted in higher biomass accumulation and final BDO titers, although differences in growth behaviour were observed during the early stages of fermentation. At lower arabinose concentrations (10–30 g L^−1^), arabinose was rapidly consumed and completely depleted within 8–12 h of cultivation. The maximum OD_600_ values achieved at initial arabinose concentrations of 10, 20, and 30 g L^−1^ were 6.17, 8.61, and 9.37, respectively. Correspondingly, BDO production occurred concomitantly with arabinose consumption, reaching final titers of 2.51, 5.01, and 10.14 g L^−1^, respectively. At an initial arabinose concentration of 40 g L^−1^, both biomass formation and BDO accumulation increased substantially. All the supplied arabinose was consumed within 14 h, resulting in a maximum OD_600_ of 12.65 and a final BDO concentration of 13.98 g L^−1^. Further increases in arabinose concentration to 50 and 60 g L^−1^ led to maximum OD_600_ values of 14.1 and 13.8, respectively, with BDO titers of 19.53 and 23.01 g L^−1^, respectively. At 60 g L^−1^, arabinose depletion required up to 40 h compared to 24 h at 50 g L^−1^, indicating slower substrate utilisation. Fermentations at 80 and 100 g L^−1^ of arabinose exhibited similar, but more pronounced effects, resulting in lower BDO productivity (data not shown). The highest BDO yields obtained at arabinose concentrations of 10, 20, 30, 40, 50, and 60 g L^−1^ were 0.26, 0.24, 0.32, 0.33, 0.40, and 0.36 g g^−1^ with corresponding productivities of 0.45, 0.42, 0.85, 1.00, 0.81, and 0.58 g L^−1^ h^−1^, respectively. These results indicate a negative effect of elevated arabinose levels on cell growth the BDO production, suggesting the presence of substrate inhibition. To address this, adaptive laboratory evolution (ALE) was employed to enhance strain tolerance and fermentation performance at elevated arabinose concentrations.

### 3.2 ALE and screening of evolved *K. pneumoniae* strains for BDO production

**Fig. 2:**
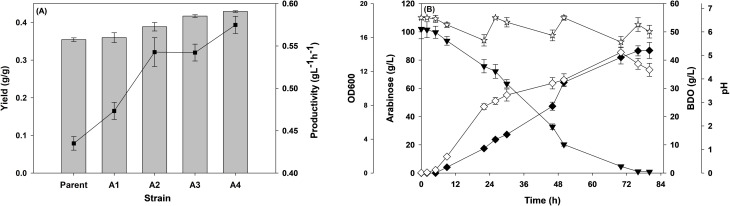
(A) Yield (g_BDO_ g_ara_^−1^ consumed) and volumetric productivity of BDO obtained from the control (parent) and ALE strains. Bars represent yield, and the line indicates productivity. (B) Time course profile of the A4 strain during pure arabinose fermentation at 100 g L^−1^. Symbols: arabinose (▼), OD_600_ (◇), BDO (◆), pH (☆).

Arabinose is the third major sugar present in LCB after glucose and xylose and the second most abundant pentose sugar after xylose.^24^ The strain was subjected to ALE using different concentrations of arabinose (40, 60, 80 and 100 g L^−1^). The various evolved strains obtained from ALE work were tested for BDO production from arabinose, and the results are summarized in SI Table 1. The TYP metrics ameliorated as the arabinose concentration was increased. The BDO production in the A4 strain (strain resulting from 100 g L^−1^ arabinose) commenced from 9 h and increased continuously till 80 h, where the highest BDO concentration of 43.8 g L^−1^ was achieved with a conversion yield of 0.43 g g^−1^ which is 86% of theoretical yield of 0.50 g g^−1^. Arabinose was completely consumed by 80 h, indicating that the cessation of BDO production was associated with substrate depletion. A comparison of BDO yield per gram of arabinose consumed and productivity of ALE strains along with the time course profile of the A4 strain for arabinose consumption, cell OD_600_ and BDO formation are shown in Fig. 2. In comparison to control, arabinose was completely metabolized in the evolved strain with better cell growth and transformation into BDO. The final cell OD_600_, BDO titer and yield were improved by 22%, 25.9% and 16.3%, respectively, as compared to the control strain. Succinic acid (SA), lactic acid (LA), formic acid (FA), acetic acid (AA) and ethanol were obtained as main byproducts, and their concentration was under 3.0 g L^−1^. The time course profiles of the strains (A1 to A3 along with the parent strain) for arabinose consumption, OD_600_, pH, BDO and byproduct formation are depicted in SI Fig. 1 and 2. The subsequent experimental work was carried out with the best BDO-accumulating A4 strain.

### 3.3 Shake flask cultivation of the A4 strain on pure sugar mixtures

**Fig. 3:**
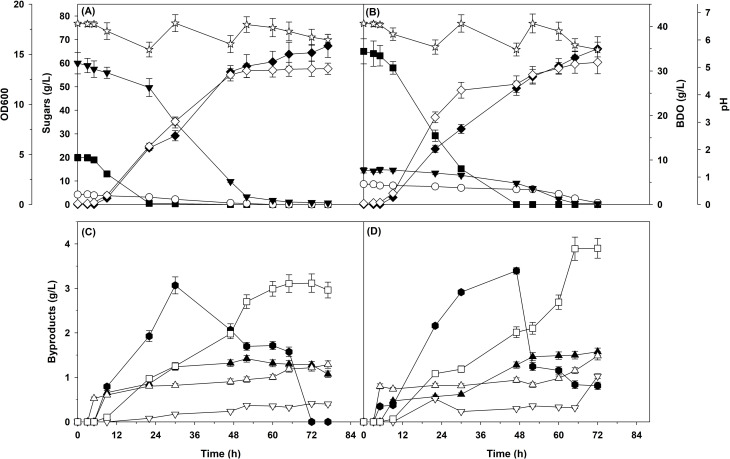
Fermentation profile of evolved *K. pneumoniae* on mixed sugars in shake flask culture showing substrate consumption, OD_600_, pH, BDO and byproducts formation: (A and C) arabinose-rich pure sugar mixture; (B and D) glucose-rich pure sugar mixture. Symbols: glucose (■), xylose (○), arabinose (▼), OD_600_ (◇), BDO (◆), pH (☆), acetoin (□), acetic acid (△), formic acid ( <svg xmlns="http://www.w3.org/2000/svg" version="1.0" width="16.000000pt" height="16.000000pt" viewBox="0 0 16.000000 16.000000" preserveAspectRatio="xMidYMid meet"><metadata> Created by potrace 1.16, written by Peter Selinger 2001-2019 </metadata><g transform="translate(1.000000,15.000000) scale(0.014583,-0.014583)" fill="currentColor" stroke="none"><path d="M400 920 l0 -40 -40 0 -40 0 0 -40 0 -40 -40 0 -40 0 0 -40 0 -40 -40 0 -40 0 0 -40 0 -40 -40 0 -40 0 0 -200 0 -200 40 0 40 0 0 -40 0 -40 80 0 80 0 0 -40 0 -40 40 0 40 0 0 -40 0 -40 80 0 80 0 0 40 0 40 40 0 40 0 0 40 0 40 80 0 80 0 0 40 0 40 40 0 40 0 0 200 0 200 -40 0 -40 0 0 40 0 40 -40 0 -40 0 0 40 0 40 -40 0 -40 0 0 40 0 40 -40 0 -40 0 0 40 0 40 -80 0 -80 0 0 -40z"/></g></svg> ), succinic acid (▲), ethanol (▽).

After the ALE work, the A4 strain was cultivated on arabinose- and glucose-rich mixtures of pure sugars to mimic the composition of biomass hydrolysates from SBP. The composition of pure sugar mixture was as follows (g L^−1^): arabinose-rich mixture – 60 arabinose, 19.9 glucose, and 4.2 xylose; glucose-rich mixture-65.0 glucose, 14.5 arabinose, and 8.6 xylose. Fig. 3 depicts the time course profiles of sugar consumption, cell OD_600_ and BDO production for both types of pure sugar mixtures. In case of arabinose-rich mixture, glucose was quickly metabolized and exhausted within 24 h. In comparison, arabinose was assimilated slowly and ∼10 g L^−1^ was consumed in 24 h. Thereafter, arabinose was metabolized rapidly, and 45 g L^−1^ of sugar was taken up in the next 24 h. Xylose was small in amount and co-metabolized with arabinose after glucose depletion. The cell growth (OD_600_) started from 6 h and a rapid increase was observed till 50 h, where the OD_600_ reached 13.4 and remained constant thereafter. The BDO production commenced from 6 h, increased continuously and the highest titer of 35.6 g L^−1^ was obtained at 77 h, and the overall BDO yield on sugars was 0.424 g g^−1^. On the hand with the glucose-rich mixture, the uptake of glucose was fast from the beginning but suppressed the utilization of two pentose sugars. All the supplied glucose was metabolized within 48 h. Fast cell growth was observed during the initial 30 h. After this period, the OD_600_ continued to increase, albeit at a slower rate, reaching a maximum value of 14.20 at 72 hours, after which it remained relatively constant. BDO production pattern was similar to the arabinose-rich mixture with the highest BDO concentration of 36.6 g L^−1^ at 77 h, and the conversion yield was 0.41 g g^−1^. Similar results on both the mixtures in terms of BDO production are quite encouraging and give hope that these two major sugars in SBP can be efficiently transformed into BDO.

### 3.4 Batch culture of the A4 strain on arabinose- and glucose-rich SBP hydrolysates

**Fig. 4:**
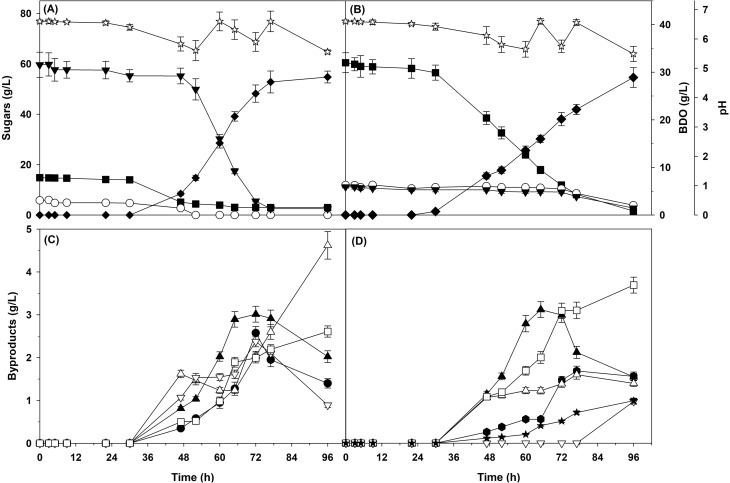
Fermentation profile of evolved *Klebsiella pneumoniae* on SBP hydrolysates in the shake flask culture showing substrate consumption, pH, BDO and byproduct formation: (A and C) SBP-derived arabinose-rich hydrolysate; (B and D) SBP-derived glucose-rich hydrolysate. Symbols: glucose (■), xylose (○), arabinose (▼), BDO (◆), pH (☆), acetoin (□), acetic acid (△), formic acid (), succinic acid (▲), citric acid (★), ethanol (▽).

In the next set of experiments, the batch cultivation of the A4 strain was repeated using arabinose- and glucose-rich hydrolysates from SBP. The arabinose-rich hydrolysate contained 59.6 g L^−1^ arabinose, 14.9 g L^−1^ glucose and 5.8 g L^−1^ xylose, while the sugar composition of the glucose-rich hydrolysate was 60.5 g L^−1^ glucose, 11.9 g L^−1^ arabinose and 11.2 g L^−1^ xylose. In case of both the hydrolysates, a strong degree of inhibition was observed as reflected by the long lag phase, leading to long fermentation periods. The cell OD could not be quantified due to interference caused by turbidity originating from biomass hydrolysate. Fig. 4 shows the sugar consumption, BDO and byproduct formation, and the pH change of the A4 strain on SBP hydrolysates. There was no significant consumption of sugars in the initial 30 h with the arabinose-rich hydrolysate. The glucose was the first sugar to metabolize at 30 h and in the next 12 h, ∼10 g L^−1^ of glucose was consumed where strain started assimilating pentose sugars. The arabinose consumption started at 48 h, and more than 90% of arabinose was exhausted in a time period of 48–72 h. The BDO accumulation was concomitant with sugar uptake and rapidly increased to 28 g L^−1^ at 77 h and became saturated beyond this time point. A similar trend was noticed with glucose-rich hydrolysates with a lag phase of 30 h, and thereafter, glucose consumption started, and significant assimilation of two pentose sugars was observed from 72 h when a large fraction of glucose was depleted. All the sugars were completely exhausted by 96 h. BDO production initiated at 30 h and accumulation continued till 96 h with a maximum titre of 28.9 g L^−1^. The conversion yield obtained with arabinose and glucose-rich hydrolysates was 0.38 and 0.39 g g^−1^, respectively. Reduced BDO metrics relative to pure sugar mixtures could be attributed to hydrolysate-derived inhibitors.

### 3.5 Fed-batch culture of the A4 strain on the mixture of pure sugars in a laboratory-scale bioreactor

**Fig. 5:**
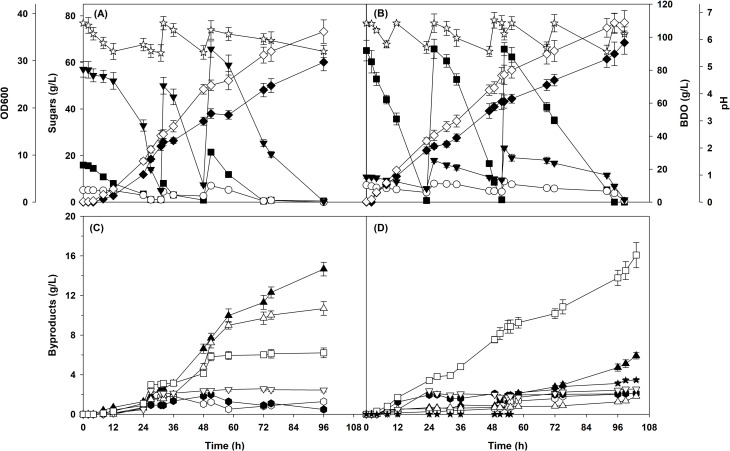
Time course profiles of sugar consumption, OD_600_, pH, BDO and byproduct formation during the fed-batch cultivation of evolved *K. pneumoniae* in the bioreactor with a mixture of pure sugars: (A and C) arabinose-rich mixture; (B and D) glucose-rich mixture. Symbols: glucose (■), xylose (○), arabinose (▼), OD_600_ (◇), BDO (◆), pH (☆), acetoin (□), acetic acid (△), formic acid (), succinic acid (▲), citric acid (★), lactic acid ( <svg xmlns="http://www.w3.org/2000/svg" version="1.0" width="17.272727pt" height="16.000000pt" viewBox="0 0 17.272727 16.000000" preserveAspectRatio="xMidYMid meet"><metadata> Created by potrace 1.16, written by Peter Selinger 2001-2019 </metadata><g transform="translate(1.000000,15.000000) scale(0.015909,-0.015909)" fill="currentColor" stroke="none"><path d="M400 840 l0 -40 -80 0 -80 0 0 -40 0 -40 -40 0 -40 0 0 -40 0 -40 -40 0 -40 0 0 -200 0 -200 40 0 40 0 0 -40 0 -40 40 0 40 0 0 -40 0 -40 80 0 80 0 0 -40 0 -40 80 0 80 0 0 40 0 40 80 0 80 0 0 40 0 40 40 0 40 0 0 40 0 40 40 0 40 0 0 200 0 200 -40 0 -40 0 0 40 0 40 -40 0 -40 0 0 40 0 40 -80 0 -80 0 0 40 0 40 -80 0 -80 0 0 -40z m160 -80 l0 -40 80 0 80 0 0 -40 0 -40 40 0 40 0 0 -200 0 -200 -40 0 -40 0 0 -40 0 -40 -80 0 -80 0 0 -40 0 -40 -80 0 -80 0 0 40 0 40 -80 0 -80 0 0 40 0 40 -40 0 -40 0 0 200 0 200 40 0 40 0 0 40 0 40 80 0 80 0 0 40 0 40 80 0 80 0 0 -40z"/></g></svg> ), ethanol (▽).

Fed-batch fermentations were conducted in a laboratory-scale bioreactor with pure sugar mixtures to further minimise substrate inhibition and improve BDO production using evolved *K. pneumoniae*. The temperature, agitation speed and aeration were controlled at 30 °C, 200 rpm and 1.0 vvm, respectively. The initial pH was 6.6 and controlled in the cyclic mode where the pH was allowed to drop till 5.5 and then brought back to 6.6 using 5 M NaOH. The fermentation was started with the mixture of pure sugars containing 57.1 g L^−1^ arabinose, 15.9 g L^−1^ glucose and 5.2 g L^−1^ xylose. Fig. 5 shows the time course profiles for sugar assimilation, OD_600_, pH, and BDO formation. The active consumption of sugars occurred after 12 h and more than 90% of sugars were consumed by 31st h, where the first batch was nearly completed. Despite arabinose being the dominant sugar, all three sugars were consumed simultaneously. A similar pattern was observed for cell OD_600_ and BDO. The significant increment in OD_600_ was noticed after 8 h, and thereafter, it continuously increased till the end where it reached 36.2. The BDO concentration accumulated by the end of first batch was 33.9 g L^−1^. The culture was fed at 31 h and ∼50 g L^−1^ of sugars were exhausted in next 17 h with improvement in BDO titer to 49.1 g L^−1^. The next feeding was done at 51 h and all the sugars were almost depleted by 96 h. The final BDO titer achieved at the end of fed-batch fermentation was 84.9 g L^−1^ with a conversion yield of 0.39 g g^−1^ and a productivity of 0.88 g L^−1^ h^−1^. Acetoin, succinic and acetic acid were obtained as main byproducts, while ethanol, formic and lactic acid were minor ones and under 3.0 g L^−1^.

Next, the fed-batch culture of the A4 strain was performed using a glucose-rich mixture containing 65.1 g L^−1^ glucose, 7.3 g L^−1^ xylose and 10.9 g L^−1^ arabinose. The trend for sugar uptake, OD_600_, metabolite production and pH are presented in Fig. 5. The fermentation on the glucose-rich mixture was faster than that on the arabinose-rich mixture, where nearly all supplied glucose was exhausted within 24 h. However, substantial amounts of xylose and arabinose were left unutilized, indicating the suppression of pentose sugars uptake in the presence of glucose. The BDO production commenced at 4 h (5.7 g L^−1^) and reached 31.4 g L^−1^ by 24 h. The first feeding was done at 27 h where glucose, xylose and arabinose concentrations were enhanced to 65.8, 8.0 and 18.0 g L^−1^, respectively. The glucose levels dropped down to 1.0 g L^−1^ with jump in BDO titer from 31.4 to 60.9 g L^−1^ at 54 h. A similar pattern was observed with xylose and arabinose. The kinetics of the first two batches in terms of sugar consumption and BDO production was quite similar. The second and last feeding of culture was done at 55 h when glucose, xylose and arabinose levels were increased to 65.7, 8.9 and 23.3 g L^−1^, respectively. The sugar consumption in the third batch was slightly slower than that in the previous two batches and it took 44 h for glucose levels to reach zero. The BDO concentration accumulated at the end of 103 h was 96.7 g L^−1^ with a conversion yield of 0.39 g g^−1^. The cell growth pattern was similar to the arabinose-rich mixture and exhibited a continuous and steady growth throughout the fermentation. The highest cell OD_600_ recorded was 38.1 towards the end of fermentation. The byproducts obtained in all experiments were acetoin, ethanol, acetic, citric, formic, lactic, and succinic acid, and among them, acetoin and succinic acid were the major ones. Table 1 shows the carbon balance of BDO production from the pure sugar mixtures.

**Table 1:** Carbon balance for BDO production using pure sugar mixtures during fed-batch fermentation by *K. pneumoniae* in a bioreactor^a^^,^^b^

Substrate & metabolites	Arabinose-rich sugar mixture			Glucose-rich sugar mixture		
	mM	C (mM)	C (%)	mM	C (mM)	C (%)
Glucose (C_6_H_12_O_6_)	239.0	1433.8	19.8	1081.8	6491.0	79.1
Xylose (C_5_H_10_O_5_)	92.0	460.2	6.4	99.4	497.0	6.1
Arabinose (C_5_H_10_O_5_)	1067.5	5337.7	73.8	244.0	1220.2	14.9
SA (C_4_H_6_O_4_)	124.2	496.7	6.9	50.1	200.3	2.4
LA (C_3_H_6_O_3_)	14.2	42.6	0.6	23.5	70.4	0.9
FA (CH_2_O_2_)	10.5	10.5	0.1	46.0	46.0	0.6
AA (C_2_H_4_O_2_)	177.8	355.7	4.9	30.2	60.4	0.7
CA (C_6_H_8_O_7_)	0.0	0.0	0.0	17.9	107.7	1.3
Ethanol (C_2_H_6_O)	53.1	106.2	1.5	55.2	110.3	1.3
Acetoin (C_4_H_8_0_2_)	70.4	281.6	3.9	182.5	729.9	8.9
BDO (C_4_H_10_O_2_)	942.8	3771.1	52.1	1074.5	4298.1	52.4
Carbon dioxide (CO_2_)^c^	2133.1	2133.1	29.5	2549.3	2549.3	31.1
Cell dry weight	456.0	456.0	6.3	480.1	480.1	5.8
Total products	7653.4			8652.4		
Carbon recovery %	105.8			105.4		

a The carbon balance calculations were made without taking carbon from the yeast extract.

b The data correspond to 96 h for arabinose-rich pure sugar and 103 h for glucose-rich pure sugar from Fig. 5.

c CO_2_ (mM) was calculated theoretically according to the formula: 2 × (BDO + acetoin) + AA + ethanol − SA. The calculation did not consider CO_2_ in the liquid and head space.

### 3.6 Fed-batch culture of the A4 strain on the arabinose-rich SBP hydrolysate in the bioreactor

**Fig. 6:**
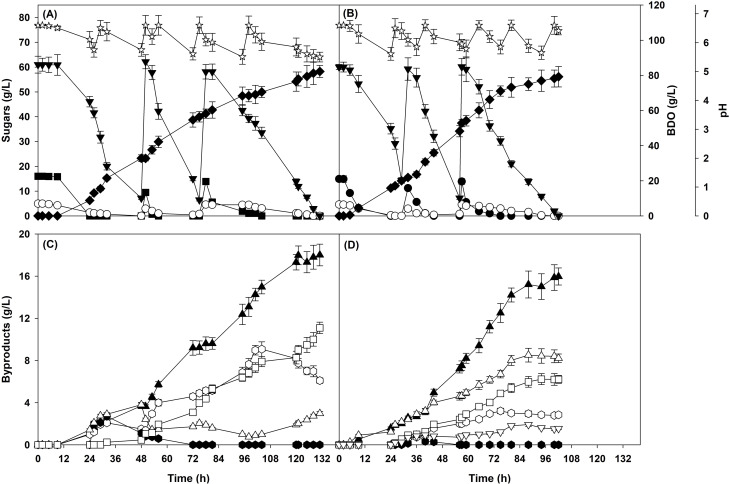
Fed-batch cultivation of evolved *K. pneumoniae* in SBP-derived arabinose-rich hydrolysates showing sugar consumption, pH, BDO and byproduct formation. (A and C) arabinose-rich hydrolysate without pasteurization, (B and D) pasteurized arabinose-rich hydrolysate. Symbols: glucose (■), xylose (○), arabinose (▼), OD_600_ (◇), BDO (◆), pH (☆), acetoin (□), acetic acid (△), formic acid (), succinic acid (▲), lactic acid (), ethanol (▽).

The fed-batch culture was repeated using arabinose- and glucose-rich hydrolysates from SBP. The composition of the arabinose-rich hydrolysate from SBP was as follows (g L^−1^): 61.0 arabinose, 16.0 glucose and 4.9 xylose. The variation in sugar consumption and BDO production during fed-batch fermentation is shown in Fig. 6. The batch fermentation starting with this non-pasteurized hydrolysate exhibited a long lag phase similar to the shake flask culture on SBP hydrolysates. After 9 h, the BDO fermentation started with slow consumption of sugars, and the uptake rate improved as the culture adapted more with the hydrolysate. At 48 h, glucose and xylose were fully depleted with a residual arabinose concentration of 7.2 g L^−1^ and BDO synthesized by this time was 32.8 g L^−1^. The BDO concentration further increased with feeding at 50 h and reached to 56.4 g L^−1^ in the next 25 h. The kinetics in this batch was better than that in the previous one as culture better acclimatized with the hydrolysate. The next feeding was done at 78 h, and this batch was slower than the previous one. All the sugars were completely consumed within 128–131 h. The BDO amassed at the end of 131 h was 82.2 g L^−1^ with a conversion yield of 0.38 g g^−1^. Acetoin, succinic and lactic acid were the major byproducts, while acetic and formic acid were the minor ones.

The fed-batch was also performed with the pasteurized arabinose-rich hydrolysate with similar hydrolysate compositions (g L^−1^): 60.0 arabinose, 14.9 glucose and 4.6 xylose. The fermentation started with gradual consumption of glucose while arabinose and xylose were assimilated slowly. At 9 h, glucose and xylose decreased to 3.21 g L^−1^, while arabinose and xylose were reduced to 53.2 and 2.9 g L^−1^, respectively, and the BDO accumulated was 4.5 g L^−1^. By 24 h, glucose was completely depleted and xylose was reduced to 0.25 g L^−1^ with an arabinose concentration of 35.2 g L^−1^ and the BDO concentration reached 15.8 g L^−1^. The culture was fed two times at 32 and 57 h, and with continued fermentation, the BDO concentration reached 79.2 g L^−1^ by 102 h, at which time all residual sugars were completely consumed. The final conversion yield of 0.38 g g^−1^ was achieved. Succinic acid was the dominant byproduct reaching ∼16 g L^−1^, while acetoin, lactic acid and acetic acid accumulated at lower concentrations (≤8 g L^−1^). Ethanol and formic acid were detected only in minor amounts. Table 2 depicts the carbon balance of BDO production from arabinose-rich hydrolysates.

**Table 2:** Carbon balance for BDO production using arabinose-rich SBP hydrolysate during fed-batch fermentation by *K. pneumoniae* in a bioreactor^a^^,^^b^^,^^d^

Substrate & metabolites	Arabinose-rich hydrolysate (non-pasteurized)			Arabinose-rich hydrolysate (pasteurized)		
	mM	C (mM)	C (%)	mM	C (mM)	C (%)
Glucose (C_6_H_12_O_6_)	218.4	1310.3	18.0	222.0	1331.8	19.1
Xylose (C_5_H_10_O_5_)	77.4	387.2	5.3	75.1	375.5	5.4
Arabinose (C_5_H_10_O_5_)	1116.5	5582.4	76.7	1051.3	5256.4	75.5
SA (C_4_H_6_O_4_)	152.5	610.0	8.4	135.2	540.9	7.8
LA (C_3_H_6_O_3_)	67.6	202.9	2.8	31.6	94.9	1.4
FA (CH_2_O_2_)	0.0	0.0	0.0	0.0	0.0	0.0
AA (C_2_H_4_O_2_)	49.5	98.9	1.4	136.7	273.4	3.9
CA (C_6_H_8_O_7_)	0.0	0.0	0.0	0.0	0.0	0.0
Ethanol (C_2_H_6_O)	0.0	0.0	0.0	33.4	66.9	1.0
Acetoin (C_4_H_8_0_2_)	125.9	503.7	6.9	70.5	281.9	4.0
BDO (C_4_H_10_O_2_)	913.7	3654.8	50.2	880.0	3520.0	50.5
Carbon dioxide (CO_2_)^c^	1976.3	1976.3	27.1	1935.9	1935.9	27.8
Cell dry weight	nd	nd	nd	nd	nd	nd
Total products	7046.6			6713.9		
Carbon recovery %	96.8			96.4		

a The carbon balance calculations were made without taking carbon from yeast extract.

b The data correspond to 131 h for non-pasteurized and 102 h for pasteurized arabinose-rich hydrolysates from Fig. 6.

c CO_2_ (mM) was calculated theoretically according to the formula: 2 × (BDO + acetoin) + AA + ethanol − SA. The calculation did not consider CO_2_ in the liquid and head space.

d This carbon balance accounts only for the major sugars in SBP hydrolysates (glucose, xylose, and arabinose) and does not include any other carbon sources. nd not determined.

A comparison between pasteurized and non-pasteurized arabinose-rich hydrolysates revealed a noticeable difference in fermentation kinetics. The first batch with the non-pasteurized hydrolysate required 48 h before the first feeding, whereas the pasteurized hydrolysate reached the first feeding stage in 32 h. This suggests that pasteurization of the hydrolysate reduced the inhibitory effects and facilitated quicker adaptation of the culture to the hydrolysate medium. Although the final BDO concentrations obtained were comparable (82.2 g L^−1^ for non-pasteurized and 79.2 g L^−1^ for pasteurized hydrolysate), the pasteurized hydrolysate enabled significantly faster fermentation progression and reduced the overall fermentation time (131 h for non-pasteurized and 102 h for pasteurized hydrolysates).

Carbon balance analysis was conducted to evaluate the carbon distribution into BDO and major metabolic byproducts. BDO consistently accounted for 50.2–52.4% of the total input carbon under all fed-batch conditions, with CO_2_ as the second largest carbon sink (27.1–31.1% C). Acetoin accumulation was notably higher in the glucose-rich pure sugar mixture (8.9% C) than in the arabinose-rich mixture (3.9% C) (Table 1). In hydrolysate fermentations, biomass could not be determined due to optical interference from the hydrolysate, and carbon recoveries were 96.4–99.1% (Tables 2 and 3). Succinic acid has a higher byproduct fraction in arabinose-rich hydrolysates (7.8–8.4% C) (Table 2) relative to glucose-rich conditions (3.0–3.6% C) (Table 3). Citric acid (CA) constituted 11.6% of input carbon in the non-pasteurized glucose-rich hydrolysate, which is over three-fold higher than the pasteurized counterpart (3.5% C), indicating substantial carbon flux diversion under inhibitor-induced metabolic stress.

**Table 3:** Carbon balance for BDO production using the glucose-rich SBP hydrolysate during fed-batch fermentation by *K. pneumoniae* in a bioreactor^a^^,^^b^^,^^d^

Substrate & metabolites	Glucose rich hydrolysate (non-pasteurized)			Glucose rich hydrolysate (pasteurized)		
	mM	C (mM)	C (%)	mM	C (mM)	C (%)
Glucose (C_6_H_12_O_6_)	1039.2	6235.5	77.7	931.3	5587.8	77.8
Xylose (C_5_H_10_O_5_)	195.8	979.0	12.2	178.0	889.9	12.4
Arabinose (C_5_H_10_O_5_)	161.6	808.1	10.1	140.8	704.0	9.8
SA (C_4_H_6_O_4_)	59.8	239.4	3.0	64.2	256.8	3.6
LA (C_3_H_6_O_3_)	25.2	75.6	0.9	23.0	68.9	1.0
FA (CH_2_O_2_)	0.0	0.0	0.0	40.0	40.0	0.6
AA (C_2_H_4_O_2_)	47.7	95.4	1.2	99.6	199.2	2.8
CA (C_6_H_8_O_7_)	155.4	932.7	11.6	41.5	249.2	3.5
Ethanol (C_2_H_6_O)	0.0	0.0	0.0	0.0	0.0	0.0
Acetoin (C_4_H_8_0_2_)	75.9	303.7	3.8	86.1	344.6	4.8
BDO (C_4_H_10_O_2_)	1026.7	4106.6	51.2	933.2	3732.9	52.0
Carbon dioxide (CO_2_)^c^	2193.0	2193.0	27.3	2074.1	2074.1	28.9
Cell dry weight	nd	nd	nd	nd	nd	nd
Total products	7946.4			6965.7		
Carbon recovery %	99.1			97.0		

a The carbon balance calculations were made without taking carbon from yeast extract.

b The data correspond to 158 h for non-pasteurized and 114 h for pasteurized glucose-rich hydrolysates from Fig. 7.

c The CO_2_ (mM) was calculated theoretically according to the formula: 2 × (BDO + acetoin) + AA + ethanol − SA. The calculation did not consider CO_2_ in the liquid and head space.

d This carbon balance accounts only for the major sugars in SBP hydrolysates (glucose, xylose, and arabinose) and does not include any other carbon sources. nd not determined.

### 3.7 Fed-batch culture of the A4 strain on the glucose-rich SBP hydrolysate in the bioreactor

After arabinose-rich hydrolysates were obtained, fed-batch culture was repeated with the glucose-rich hydrolysate containing 62.9 g L^−1^ glucose, 10.4 g L^−1^ xylose and 8.6 g L^−1^ arabinose. The lag phase with the glucose-rich hydrolysate was much longer than that with the arabinose-rich hydrolysate, and lasted for 29 h where hardly any sugar was consumed. Fig. 7 provides the time course profiles for sugar utilization and BDO formation at specific intervals. The sugar assimilation started after 29 h, and the glucose concentration dropped to zero, while the xylose and arabinose concentrations were reduced to 5.8 and 6.2 g L^−1^ at 56 h. The culture was fed at two time points, 75 and 121 h. After both the feedings, the strain took 35–45 h to assimilate the total sugars provided in the range of 75–85 g L^−1^. In all the batches, the active consumption of xylose and arabinose occurred only after a major fraction of glucose was metabolized. The BDO level accumulated at 56 h was 30.6 g L^−1^ and after two feedings at 75 and 121 h, it increased to 68.9 and 92.4 g L^−1^, respectively. The overall conversion yield was 0.38 g g^−1^ with a productivity of 0.58 g L^−1^ h^−1^. Surprisingly, citric acid was obtained as a major product (29.8 g L^−1^) in case of the glucose-rich hydrolysate, while the concentration of the other byproducts (acetoin, acetic, lactic and succinic acid) was not more than 8.0 g L^−1^.

**Fig. 7:**
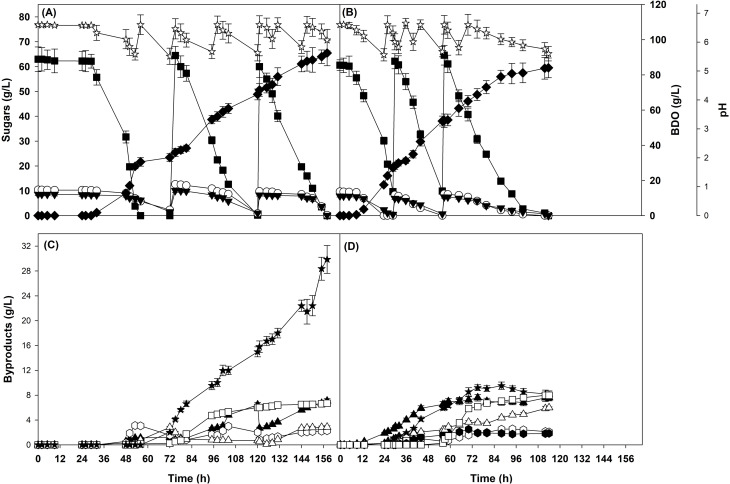
Variation in sugar consumption, OD_600_, pH, BDO, and byproduct formation (acetoin, acetic, formic, succinic, lactic, and citric acid and ethanol) during the fed-batch cultivation of evolved *K. pneumoniae* in the bioreactor using SBP-derived glucose-rich hydrolysate. (A and C) glucose-rich hydrolysate without pasteurization, (B and D) pasteurized glucose-rich hydrolysate. Symbols: glucose (■), xylose (○), arabinose (▼), OD_600_ (◇), BDO (◆), pH (☆), acetoin (□), acetic acid (△), formic acid (), succinic acid (▲), citric acid (★), lactic acid (), ethanol (▽).

Similarly, the fed-batch culture was repeated with a pasteurized glucose-rich hydrolysate containing 60.6 g L^−1^ glucose, 9.7 g L^−1^ xylose and 7.9 g L^−1^ arabinose. The fermentation exhibited a short lag phase compared to the non-pasteurized glucose-rich hydrolysate. At 13 h, the glucose concentration decreased to 48.3 g L^−1^, while xylose and arabinose concentrations were reduced to 7.0 and 6.8 g L^−1^, respectively, and the BDO produced was 3.6 g L^−1^. Active sugar assimilation occurred afterwards and the BDO concentration increased to 27.2 g L^−1^ at 29 h. The culture was first fed at 30 h and the sugars supplied in this feed were rapidly assimilated without any additional lag phase, and the second feed was done at 57 h. During this stage of fermentation, glucose was gradually consumed along with xylose and arabinose. At 88 h, glucose, xylose and arabinose concentrations were reduced to 13.9, 2.2 and 3.1 g L^−1^, respectively, while the BDO concentration reached 78.9 g L^−1^. All the residual sugars were exhausted by 114 h. The final BDO concentration achieved was 84.0 g L^−1^ at the end of fermentation with a conversion yield of 0.39 g g^−1^. The byproduct profile during fermentation with the pasteurized glucose-rich hydrolysate was similar to that observed with the non-pasteurized hydrolysate. The concentrations of byproducts such as acetoin, citric acid, acetic acid, lactic acid and succinic acid remained relatively low throughout the fermentation, and were under 8 g L^−1^. Table 3 shows the carbon balance of BDO production from the glucose-rich hydrolysate.

A comparison between pasteurized and non-pasteurized glucose-rich hydrolysates revealed a clear difference in fermentation kinetics. In the case of non-pasteurized hydrolysate, the first feeding was carried out at 75 h following a prolonged lag phase of 29 h, whereas with pasteurized hydrolysate the culture reached the feeding stage much earlier at 30 h. These results indicate that pasteurization enabled faster initiation of fermentation and improved the overall substrate assimilation during fed-batch cultivation.

## 4. Discussion

White biotechnology represents a key pillar of the bioeconomy, and the expanded use of microbial cell factories offers significant opportunities for establishing a sustainable bio-based industrial framework.^25^ The finite availability of fossil fuels has accelerated the development of biological approaches for the sustainable production of chemicals from renewable resources. Among the factors influencing the economic feasibility of bioprocesses, feedstock cost is one of the most critical parameters for sustainable bioproduct manufacturing. Biogenic residues generated from agro-industrial sectors represent promising substrates for the fermentative production of chemicals and can therefore contribute to both commercial viability and environmental sustainability.^2^ Consequently, the valorization of such biowastes is gaining increasing global attention and has emerged as an important research focus. In this context, fermentation plays a crucial role, as renewable feedstocks are abundant and inexpensive sources of fermentable sugars that can be converted into chemical building blocks through microbial processes.^26,27^ BDO is an important industrial chemical whose molecular structure enables a wide range of chemical transformations, allowing its conversion into various value-added derivatives. Microbial production of BDO from renewable feedstocks offers several advantages in terms of sustainability and environmental benefits, including the potential to mitigate global warming. However, fermentative BDO production was historically replaced by a less expensive fossil-based route involving hydrocarbon processing under extreme temperature and pressure conditions, an energy-intensive and environmentally detrimental process.^17,18,28,29^ Despite the advantages of microbial production and the high BDO titers reported in several studies, large-scale implementation of biotechnological BDO production has not yet been fully realized due to multiple challenges, including high feedstock costs, expensive product recovery, and issues related to isomer purity. In fact, substrate cost alone can account for up to 50% of the total manufacturing expense, thereby limiting process scalability.^2,19^ Therefore, the present study aimed to develop a cost-effective process for BDO production using C5 sugars (arabinose and xylose) and a C6 sugar (glucose) derived from SBP by a strain of *K. pneumoniae*.

The microbial production of alcohols represents a highly stressful physiological condition for the producing organisms. One of the primary stresses arises from the substrates used for cultivation, which often contain high concentrations of sugars.^30^ In a recent study, Narisetty *et al.* (2022) have reported BDO production from both pure and crude arabinose derived from SBP using an *Enterobacter ludwigii* strain.^20^ However, the obtained BDO titers were substantially lower compared with production metrics reported for glucose- and xylose-based fermentations in the literature.^23,31^ Similarly, in the present study, substrate inhibition was observed at arabinose concentrations above 50 g L^−1^ (Fig. 1). ALE is frequently employed for the efficient utilization of substrate, thereby improving the metabolite production. In the present work, the study was initiated by characterizing arabinose-induced substrate limitation and subsequently subjecting *K. pneumoniae* to ALE in order to improve arabinose tolerance and BDO production. ALE has emerged as a powerful and widely used strategy for enhancing the microbial fitness bestowed through the accumulation of beneficial mutations that allow the evolved strains to outcompete their parental counterparts and dominate the population. This approach has significant potential to alter microbial responses to various feedstocks and to facilitate the development of robust cell factories capable of improved chemical conversion to desired products, which is often difficult to achieve through purely rational metabolic engineering strategies.^32,33^ As shown in SI Table 1, the evolved strains demonstrated substantial improvements in sugar consumption, cell growth, and BDO accumulation. The cell OD_600_, BDO titer and yield in the evolved strain enhanced from 12, 34.8 g L^−1^ and 0.36 g g^−1^ to 13.4, 43.8 g L^−1^ and 0.43 g g^−1^, respectively, in comparison to control parent strain. Similar to our work, there are several studies in the literature where ALE has led to significant improvement in tolerance to high levels of substrate, utilization of substrate, and metabolite production (Table 4).^30,34^ However, studies focusing specifically on arabinose as a carbon source and BDO as the target product remain scarce.

**Table 4:** Summary of the ALE work for effective substrate utilization and enhanced product formation

Microorganism	Selection pressure for ALE	Desirable features of the evolved strain	Reference
*Saccharomyces cerevisiae*	Yeast cultivation at 20 and 50 g L^−1^ lactose	Rapid lactose (two-fold faster) fermentation and enhanced ethanol production by 30%	47
*Saccharomyces cerevisiae*	40 days chemostat cultivation on xylose–arabinose mixtures	1.5-Fold increment in consumption of pentose sugars with reduced fermentation time	48
*Zymomonas mobilis*	Adaptation of recombinant *Z. mobilis* for ∼300 generations at 50 and 100 g L^−1^ xylose	Significant improvement in xylose uptake rate (∼50–250%) and ethanol production (∼60–80%)	36
*Kluyveromyces marxianus*	Continuously increasing lactose concentration from whey permeate for 65 days	Higher utilization of lactose (>80% w/v) with enhanced ethanol levels (80 g L^−1^)	35
*Saccharomyces cerevisiae*	Enhancing galactose level from 20 to 80 g L^−1^	Improved ethanol titer from 8.9 to 20 g L^−1^ with conversion yield of 0.44 g g^−1^	49
*Zymomonas mobilis*	Exposing increasing xylose concentrations up to 100 g L^−1^ over a period of 200 days	Enhanced xylose assimilation, growth, co-utilization of mixed sugars, ethanol production, and reduced xylitol production	37
*K. pneumoniae*	Gradual adaptation of *K. pneumoniae* to increasing arabinose concentrations from 40–100 g L^−1^	Improved arabinose utilization and BDO titer at 100 g L^−1^; 22% increase in cell biomass, and 26% increase in BDO yield	This study

For instance, Saini *et al.* (2017) subjected the dairy yeast *Kluyveromyces marxianus* to ALE using progressively increasing lactose concentrations derived from whey permeate.^35^ The adapted strain exhibited higher β-galactosidase activity and was able to efficiently ferment whey permeate containing 200 g L^−1^ lactose, utilizing more than 80% (w/v) of the sugar and producing higher ethanol concentrations (80 g L^−1^) compared with the parental strain. In another work, Dunn and Rao (2015) subjected a recombinant *Zymomonas mobilis* strain to ALE on xylose and arabinose, yielding evolved strains with significantly improved pentose utilisation (∼50–250%) and ethanol production (∼60–80%). High-throughput sequencing revealed mutations in the xylose reductase gene that reduced xylitol accumulation and improved xylose fermentation. Additionally, decreased glyceraldehyde-3-phosphate dehydrogenase activity and increased transketolase activity contributed to improved arabinose metabolism in *Z. mobilis*. Further improvements in pentose fermentation were associated with mutations in the native sugar transporter (Glf) and AddB protein, which enhanced plasmid stability and reduced cell aggregation.^36^ Similarly, Sarkar *et al.* (2020) subjected a recombinant xylose-fermenting *Z. mobilis* strain to ALE in media containing progressively increasing xylose concentrations up to 100 g L^−1^ over a period of 200 days. The evolved strain showed altered phenotypes, including enhanced xylose assimilation, growth, co-utilization of mixed sugars, ethanol production, and reduced xylitol production in comparison to the parental strain. Next-generation sequencing identified mutations in several genes, including *xylA*, *tklB*, *glf*, *pdc*, *amtB*, *gcrA*, *gyrB2*, and *kdsB*, which were associated with the improved phenotypes.^37^ A detailed summary of various ALE reports on substrate utilization is given in Table 4.

Although microbial BDO production offers clear sustainability advantages, its industrial viability remains constrained by high production costs, with substrate alone accounting for a significant fraction of total expenditure. Therefore, the use of inexpensive carbon sources is essential to improve the economic feasibility of the process.^38^ LCB is a mixture of hexose and pentose sugars; therefore, industrial strains that can effectively and simultaneously metabolize all these sugars are highly desired.^9^ SBP represents an inexpensive and abundant feedstock, with more than 50% of its dry mass composed of fermentable sugars (C5 and C6), predominantly glucose and arabinose. Several studies have reported the utilization of cellulosic glucose for the production of various metabolites, including BDO.^18^ However, despite their abundance in lignocellulosic hydrolysates, pentose sugars, particularly arabinose, remain underutilized in biotechnological processes.^24^ SBP contains comparable amounts of glucose and arabinose,^39^ and in the present study, both of these sugars were integrated for fermentative BDO production. This will allow maximal utilization of fermentable sugars present in SBP and could improve the process economics. One of the major challenges associated with arabinose utilization is that many industrially relevant microbial cell factories lack the metabolic capability to efficiently assimilate this sugar.^24^ An advantageous feature of the *K. pneumoniae* strain used in this study is its ability to grow on pentose sugars, including xylose and arabinose, while efficiently accumulating metabolites. A survey of the literature shows that there are a handful of studies on BDO accumulation from arabinose.^5,20,21,40,41^ A study by Saha and Bothast reported that a newly isolated *Enterobacter cloacae* strain produced 7.0–34.4 g L^−1^ BDO from 20–100 g L^−1^ arabinose with yields ranging from 0.34–0.38 g g^−1^. However, fermentation performance declined as the substrate concentration increased. Cultures containing 100 g L^−1^ arabinose required up to 72 h for completion, whereas lower concentrations required shorter fermentation times (24 h for 10–30 g L^−1^ and 32 h for 40–50 g L^−1^).^40^ A similar trend was observed in the present study, where fermentation performance began to slow down from 50 g L^−1^ arabinose and showed a pronounced effect at 60 g L^−1^, which motivated the implementation of ALE. The results obtained in the present study are substantially higher than those previously reported. The evolved strain demonstrated markedly improved performance for BDO production at both shake-flask and bioreactor scales. When cultivated on pure arabinose, the BDO titer obtained in this work was nearly twice that reported previously (84.9 g L^−1^*vs.* 42.9 g L^−1^). SI Table 2 shows a comparison of biomass formation, and TYP metrics of BDO production under various experimental conditions. These findings highlight the potential of arabinose as a viable carbon source for BDO production and demonstrate the suitability of SBP as a promising feedstock in which both major sugars can be effectively utilized for BDO biomanufacturing. Similar to several studies that have employed LCB hydrolysates for BDO production,^20,23^ inhibitory effects were also observed during fermentation with SBP hydrolysate, as indicated by a prolonged lag phase. The inhibition may be due to the presence of fermentation inhibitors (organic acids, furan derivatives, and phenolic compounds) released during SBP pretreatment alongside the fermentable sugars, which can impact the subsequent fermentation process *via* toxic effects on microorganisms and enzymes.^5^

In the present study, pasteurization of SBP hydrolysates resulted in a noticeable improvement in fermentation performance. Fermentations carried out with pasteurized hydrolysates showed shorter lag phases and earlier feeding during fed-batch operation compared to non-pasteurized hydrolysates. This led to faster substrate assimilation and improved overall fermentation kinetics. Consequently, fermentations using pasteurized arabinose-rich and glucose-rich hydrolysates achieved comparable BDO titers (79.2 and 84.0 g L^−1^) and yields (0.38 and 0.39 g g^−1^) to those obtained with non-pasteurized hydrolysates (82.2 and 92.4 g L^−1^), while significantly reducing the overall fermentation time and improving the productivity. The improved fermentation kinetics observed with pasteurized hydrolysates may be attributed to the heat-induced mitigation of inhibitory effects within the medium. Even though no sugar degradation products were detected in the hydrolysates, the inhibitory pressures may arise from phenolic compounds generated during acid hydrolysis, as well as residual oxidizing species originating from the hydrogen peroxide pretreatment, both of which can impose oxidative stress on microbial cells and prolong the lag phase. While direct quantitative analysis of inhibitors before and after pasteurization was not performed, the enhanced fermentation kinetics aligned with well-documented thermochemical degradation mechanisms. Thermal processing accelerates the degradation and oxidation of free phenolic compounds.^56^ Silva *et al*. (2025) empirically demonstrated that thermal processing of enzyme hydrolysed seriguela pulp over 40 °C to 70 °C for over 120 minutes resulted in significant losses of total free phenolic compounds *via* first-order degradation kinetics.^56^ At 70 °C incubation of hydrolysed pulp, they found a total loss of 70% total phenolics compounds by 30 min. Similarly, at 70 °C under near-neutral pH conditions, the spontaneous decomposition of residual H_2_O_2_ is promoted, as the decomposition rate increases with both temperature and pH.^57^ Consistent with these observations, Amraoui and colleagues reported a significant difference between the performance of *E. ludwigii* when cultured on non-detoxified and detoxified xylose-rich hemicellulosic hydrolysate derived from sugarcane bagasse. In the detoxified hydrolysate, the BDO titer and yield reached 63.5 g L^−1^ and 0.36 g g^−1^, respectively, whereas these values decreased to 32.7 g L^−1^ and 0.33 g g^−1^ when the non-detoxified hydrolysate was used.^23^ Additionally, Lee *et al.* (2015) and Joo *et al.* (2016) investigated the impact of organic acids, furan derivatives and phenolic compounds on cell growth and BDO production by *Enterobacter aerogenes*.^42,43^ Their studies demonstrated that these compounds had a negative effect on cell growth and the biosynthesis of BDO, and most toxic effects were observed with phenolic compounds. Similar to the findings reported by Narisetty *et al.*,^20^ the carbon balance analysis in this study indicates that the majority of the supplied carbon was partitioned between BDO production (∼50%) and carbon dioxide (CO_2_) evolution (∼30%). The remaining fraction of the carbon flux was diverted toward the formation of byproducts, accounting for the remaining carbon loss.

Table 5 presents the comparison of microbial BDO production using both pure and crude sugars, including glucose, xylose, and arabinose. The Table summarizes the findings of the present study alongside those previously reported in the literature. Most BDO-producing microorganisms efficiently convert glucose into BDO; however, many of these strains either lack the ability to utilize pentose sugars or exhibit limited capacity to convert xylose and arabinose into BDO.^44^ In comparison with several reported strains, *K. pneumoniae* demonstrated superior BDO production when cultivated on pentose sugars, particularly arabinose. Nevertheless, when compared with BDO accumulation on glucose (as shown in Table 5), BDO titer from arabinose in the present study was lower and required a longer fermentation time. This performance gap could potentially be reduced through targeted metabolic engineering strategies, as demonstrated in several previous studies. For instance, Li *et al.* employed an *E. cloacae* strain for BDO production using a mixture of glucose and xylose, the two major sugars present in LCB.^45^ The strain was metabolically engineered through overexpression of BDO dehydrogenase and galactose permease, elimination of carbon catabolite repression, and reduction of by-product formation such as lactic and succinic acids. As a result, the engineered strain produced significantly high levels of BDO (115–155 g L^−1^) with yields of 0.47–0.49 g g^−1^ and productivities of 2.0–4.0 g L^−1^ h^−1^ when cultivated on glucose–xylose mixtures as well as corn stover hydrolysate. Similarly, Kim and team metabolically engineered *E. aerogenes* strain to amplify the BDO pathway, improve the glucose uptake rate and reduce the byproduct formation. The engineered cell factory displayed substantial improvement as compared to control strain with the BDO titer, yield, and productivity of 114.3 g L^−1^, 0.44 g g^−1^ and 1.49 g L^−1^ h^−1^, respectively.^9^ Although the *K. pneumoniae* strain used in the present study demonstrated improved performance, the production metrics reported in that study remained comparatively higher. On the other hand, some studies have reported lower BDO production from glucose compared with the arabinose-based production achieved in the present work. For example, Ling *et al.* (2017) achieved BDO titer of 52.5 g L^−1^ with a conversion yield of 0.42 g g^−1^ and a productivity of 0.88 g L^−1^ h^−1^ during fed-batch fermentation using glucose and xylose from corncob hydrolysates.^46^ Overall, the evolved *K. pneumoniae* strain demonstrates strong potential as a microbial cell factory for LCB-based BDO production, owing to its ability to efficiently utilize glucose, xylose, and arabinose.

**Table 5:** Summary of BDO bioproduction from pure sugars and sugar-rich hydrolysates

Microorganism	Feedstock	Fermentation mode	BDO			Reference
			Titer (g L^−1^)	Yield (g g^−1^)	Productivity (g L^−1^ h^−1^)	
*Klebsiella pneumoniae*	Pure xylose	Fed-batch	42.7	0.47	1.17	50
*Enterobacter cloacae*	Xylose solution	Fed-batch	81.4	0.39	0.72	51
*Klebsiella pneumoniae*	Pure xylose	Batch	38.6	—	0.62	52
*Bacillus vallismortis* B-14891	Pure xylose	Batch	26.5	0.32	1	53
*Enterobacter ludwigii*	Pure xylose	Fed-batch	71.1	0.40	0.94	23
*Enterobacter ludwigii*	Xylose rich SCB hydrolysate	Fed-batch	63.5	0.36	0.84	23
*Enterobacter cloacae*	Pure arabinose	Batch	34.4	0.34	0.48	40
*Enterobacter aerogenes*	Pure arabinose	Batch	7.3	0.33	—	41
*Enterobacter ludwigii*	Pure arabinose	Fed-batch	42.9	0.31	0.60	20
*Enterobacter ludwigii*	Arabinose rich SBP hydrolysate	Fed-batch	35.5	0.29	0.49	20
*Bacillus licheniformis*	Pure glucose	Fed-batch	115.7	0.47	2.4	54
*Bacillus licheniformis*	Pure glucose	Fed-batch	144.7	0.40	1.14	55
*Enterobacter cloacae*	Pure glucose and xylose	Fed-batch	152.0	0.49	3.50	45
*Enterobacter cloacae*	Corn stover hydrolysate	Fed-batch	119.4	0.48	2.30	45
*Enterobacter cloacae*	Corncob hydrolysate	Fed-batch SHF	55.7	0.23	0.93	46
*Enterobacter cloacae*	Sugarcane bagasse hydrolysate	Fed-batch SHF	114.3	0.44	1.49	9
*Enterobacter ludwigii*	Brewers spent grain hydrolysate	Fed-batch	118.5	0.43	1.65	31
*Klebsiella pneumoniae* PX14	Unpretreated bamboo	Fed-batch SSF	43.5	—	0.36	38
*Klebsiella pneumoniae*	Arabinose rich sugar mixture	Fed-batch	84.9	0.39	0.88	This study
*Klebsiella pneumoniae*	Glucose rich sugar mixture	Fed-batch	96.7	0.39	0.94	This study
*Klebsiella pneumoniae*	Arabinose rich hydrolysate (non-pasteurized)	Fed-batch	82.2	0.38	0.63	This study
	Arabinose rich hydrolysate (pasteurized)		79.21	0.38	0.78	
*Klebsiella pneumoniae*	Glucose rich SBP hydrolysate (non-pasteurized)	Fed-batch	92.4	0.38	0.58	This study
	Glucose rich hydrolysate (pasteurized)		84.00	0.39	0.74	

## 5. Conclusions

Lignocellulosic materials are gaining increasing prominence due to their direct role in replacing and reducing the consumption of fossil-based resources, and show immense potential to serve as feedstocks for sustainable circular biorefineries. One of the advantageous features of fermentative route is that it also gives the possibility of valorizing crude renewable sources into industrial products. The full potential can be realized if maximum renewable carbon from bioresource could be utilized and diverted towards the desired product. In the present study, the valorization of the two major sugars present in SBP, arabinose and glucose, was investigated for the fermentative production of BDO using an evolved *K. pneumoniae* strain as the microbial cell factory. The TYP metrics achieved with pure sugars and sugar-rich SBP hydrolysates were found to be comparable, demonstrating the feasibility of utilizing SBP-derived sugars for BDO production. Future studies will focus on whole-genome sequencing to elucidate the genetic basis of the improved phenotype observed after adaptive laboratory evolution. Additionally, further efforts are required to enhance the economic feasibility of the process, and future research will focus on advanced metabolic engineering and process optimization strategies to further improve BDO production and move toward industrial implementation.

## Conflicts of interest

The authors declare that they have no known conflicting financial interests or personal relationships that could have appeared to influence the work reported in this paper.

## Supplementary Material

SE-010-D6SE00474A-s001

## Data Availability

All data generated or analyzed during this study are included in this published article. Supplementary information (SI): tables comparing BDO production metrics for the strains obtained through ALE, as well as fed-batch fermentations using pure sugar mixtures and SBP hydrolysates. It also includes figures showing time-course fermentation data and byproduct profiles for all ALE-derived strains cultivated on 100 g L^−1^ arabinose. See DOI: https://doi.org/10.1039/d6se00474a.

## References

[cit1] Koutinas A. A., Vlysidis A., Pleissner D., Kopsahelis N., Lopez Garcia I., Kookos I. K., Papanikolaou S., Kwan T. H., Lin C. S. K. (2014). Valorization of industrial waste and by-product streams via fermentation for the production of chemicals and biopolymers. Chem. Soc. Rev..

[cit2] Karayannis D., Angelou N., Vasilakis G., Charisteidis I., Litinas A., Papanikolaou S. (2025). A non-aseptic bioprocess for production and recovery of 2,3-butanediol via conversion of crude glycerol and corn steep liquor at pilot-scale. Carbon Resour. Convers..

[cit3] Agrawal D., Awani K., Nabavi S. A., Balan V., Jin M., Aminabhavi T. M., Dubey K. K., Kumar V. (2023). Carbon emissions and decarbonisation: The role and relevance of fermentation industry in chemical sector. Chem. Eng. J..

[cit4] Becker J., Liebal U. W., Phan A. N., Ullmann L., Blank L. M. (2023). Renewable carbon sources to biochemicals and -fuels: contributions of the smut fungi Ustilaginaceae. Curr. Opin. Biotechnol..

[cit5] Marisutti E., Viegas B. M., Rodrigues N. P., Ayub M. A. Z., Rossi D. M. (2024). Characterization and treatments in soybean hull for 2,3-Butanediol production using *Klebsiella pneumoniae BLh-1* and *Pantoea agglomerans BL1*. Renewable Energy.

[cit6] Guo Z., Yan N., Lapkin A. A. (2019). Towards circular economy: integration of bio-waste into chemical supply chain. Curr. Opin. Chem. Eng..

[cit7] Morseletto P. (2020). Targets for a circular economy. Resour. Conserv. Recycl..

[cit8] Ptak M., Skowrońska A., Pińkowska H., Krzywonos M. (2022). Sugar Beet Pulp in the Context of Developing the Concept of Circular Bioeconomy. Energies.

[cit9] Kim D. G., Yoo S. W., Kim M., Ko J. K., Um Y., Oh M. K. (2020). Improved 2,3-butanediol yield and productivity from lignocellulose biomass hydrolysate in metabolically engineered Enterobacter aerogenes. Bioresour. Technol..

[cit10] Dhodduraj K., Narisetty V., Nabavi S. A., Saldivar R. P., Coulon F., Agrawal D., Maity S. K., Balan V., Kumar V. (2026). Beets beyond sugar: Potential and limitations of sugar beet pulp as a feedstock for biorefineries. Ind. Crops Prod..

[cit11] Berlowska J., Cieciura-Wloch W., Kalinowska H., Kregiel D., Borowski S., Pawlikowska E., Binczarski M., Witonska I. (2018). Enzymatic Conversion of Sugar Beet Pulp: A Comparison of Simultaneous Saccharification and Fermentation and Separate Hydrolysis and Fermentation for Lactic Acid Production. Food Technol. Biotechnol..

[cit12] Glaser S. J., Abdelaziz O. Y., Demoitié C., Galbe M., Pyo S.-H., Jensen J. P., Hatti-Kaul R. (2024). Fractionation of sugar beet pulp polysaccharides into component sugars and pre-feasibility analysis for further valorisation. Biomass Convers. Biorefin..

[cit13] EC , EU Agricultural Outlook for Markets, Income and Environment 2020–2030, Publications Office of the European Union, 2020, 10.2762/252413

[cit14] Puligundla P., Mok C. (2021). Valorization of sugar beet pulp through biotechnological approaches: recent developments. Biotechnol. Lett..

[cit15] Rana A. K., Gupta V. K., Newbold J., Roberts D., Rees R. M., Krishnamurthy S., Thakur V. K. (2022). Sugar beet pulp: Resurgence and trailblazing journey towards a circular bioeconomy. Fuel.

[cit16] 2 3 Butanediol market size was USD 275.5 million in 2023, https://www.cognitivemarketresearch.com/2-3-butanediol-market-report (accessed March 7, 2026)

[cit17] Maina S., Prabhu A. A., Vivek N., Vlysidis A., Koutinas A., Kumar V. (2022). Prospects on bio-based 2,3-butanediol and acetoin production: Recent progress and advances. Biotechnol. Adv..

[cit18] Bai Y., Feng H., Liu N., Zhao X. (2023). Biomass-Derived 2,3-Butanediol and Its Application in Biofuels Production. Energies.

[cit19] Tinôco D., Borschiver S., Coutinho P. L., Freire D. M. G. (2021). Technological development of the bio-based 2,3-butanediol process. Biofuel Bioprod. Biorefining.

[cit20] Narisetty V., Narisetty S., Jacob S., Kumar D., Leeke G. A., Chandel A. K., Singh V., Srivastava V. C., Kumar V. (2022). Biological production and recovery of 2,3-butanediol using arabinose from sugar beet pulp by *Enterobacter ludwigii*. Renewable Energy.

[cit21] Cortivo P. R. D., Machado J., Hickert L. R., Rossi D. M., Ayub M. A. Z. (2019). Production of 2,3-butanediol by *Klebsiella pneumoniae* BLh-1 and *Pantoea agglomerans* BL1 cultivated in acid and enzymatic hydrolysates of soybean hull. Biotechnol. Prog..

[cit22] Dhodduraj K. , Narisetty V. and Kumar V., Unlocking Sugar Beet Pulp *via* Novel Hydrogen Peroxide-Tween 80 and Polypropylene Glycol Pretreatments: Mechanistic Insights and Comparative Techno-Economic Analysis, [Unpublished Results], 2026

[cit23] Amraoui Y., Narisetty V., Coulon F., Agrawal D., Chandel A. K., Maina S., Koutinas A., Kumar V. (2021). Integrated Fermentative Production and Downstream Processing of 2,3-Butanediol from Sugarcane Bagasse-Derived Xylose by Mutant Strain of *Enterobacter ludwigii*. ACS Sustain. Chem. Eng..

[cit24] Kumar V., Agrawal D., Bommareddy R. R., Islam M. A., Jacob S., Balan V., Singh V., Thakur V. K., Navani N. K., Scrutton N. S. (2024). Arabinose as an overlooked sugar for microbial bioproduction of chemical building blocks. Crit. Rev. Biotechnol..

[cit25] Rusu A. V., Trif M., Rocha J. M. (2023). Microbial Secondary Metabolites via Fermentation Approaches for Dietary Supplementation Formulations. Molecules.

[cit26] Fiorentino G., Ripa M., Ulgiati S. (2017). Chemicals from biomass: technological versus environmental feasibility. A review. Biofuel Bioprod. Biorefining.

[cit27] Zuiderveen E. A. R., Kuipers K. J. J., Caldeira C., Hanssen S. V., van der Hulst M. K., de Jonge M. M. J., Vlysidis A., van Zelm R., Sala S., Huijbregts M. A. J. (2023). The potential of emerging bio-based products to reduce environmental impacts. Nat. Commun..

[cit28] Białkowska A. M. (2016). Strategies for efficient and economical 2,3-butanediol production: new trends in this field. World J. Microbiol. Biotechnol..

[cit29] Gräfje H. , Körnig W., Weitz H.-M., Reiß W., Steffan G., Diehl H., Bosche H., Schneider K., Kieczka H. and Pinkos R., Butanediols, Butenediol, and Butynediol, Ullmann's Encyclopedia of Industrial Chemistry, 2019, pp. 1–12, 10.1002/14356007.a04_455.pub2

[cit30] Mavrommati M., Daskalaki A., Papanikolaou S., Aggelis G. (2022). Adaptive laboratory evolution principles and applications in industrial biotechnology. Biotechnol. Adv..

[cit31] Amraoui Y., Prabhu A. A., Narisetty V., Coulon F., Kumar Chandel A., Willoughby N., Jacob S., Koutinas A., Kumar V. (2022). Enhanced 2,3-Butanediol production by mutant *Enterobacter ludwigii* using Brewers' spent grain hydrolysate: Process optimization for a pragmatic biorefinery loom. Chem. Eng. J..

[cit32] Shepelin D., Hansen A. S. L., Lennen R., Luo H., Herrgård M. J. (2018). Selecting the Best: Evolutionary Engineering of Chemical Production in Microbes. Genes.

[cit33] Sandberg T. E., Salazar M. J., Weng L. L., Palsson B. O., Feist A. M. (2019). The emergence of adaptive laboratory evolution as an efficient tool for biological discovery and industrial biotechnology. Metab. Eng..

[cit34] Wang G., Li Q., Zhang Z., Yin X., Wang B., Yang X. (2023). Recent progress in adaptive laboratory evolution of industrial microorganisms. J. Ind. Microbiol. Biotechnol..

[cit35] Saini P., Beniwal A., Kokkiligadda A., Vij S. (2017). Evolutionary adaptation of Kluyveromyces marxianus strain for efficient conversion of whey lactose to bioethanol. Process Biochem..

[cit36] Dunn K. L., Rao C. V. (2015). High-throughput sequencing reveals adaptation-induced mutations in pentose-fermenting strains of *Zymomonas mobilis*. Biotechnol. Bioeng..

[cit37] Sarkar P., Mukherjee M., Goswami G., Das D. (2020). Adaptive laboratory evolution induced novel mutations in *Zymomonas mobilis* ATCC ZW658: a potential platform for co-utilization of glucose and xylose. J. Ind. Microbiol. Biotechnol..

[cit38] Li Y. Q., Wang M. J., Gan X. F., Luo C. B. (2023). Cleaner 2,3-butanediol production from unpretreated lignocellulosic biomass by a newly isolated *Klebsiella pneumoniae PX14*. Chem. Eng. J..

[cit39] Cárdenas-Fernández M., Bawn M., Hamley-Bennett C., Bharat P. K. V., Subrizi F., Suhaili N., Ward D. P., Bourdin S., Dalby P. A., Hailes H. C., Hewitson P., Ignatova S., Kontoravdi C., Leak D. J., Shah N., Sheppard T. D., Ward J. M., Lye G. J. (2017). An integrated biorefinery concept for conversion of sugar beet pulp into value-added chemicals and pharmaceutical intermediates. Faraday Discuss..

[cit40] Saha B. C., Bothast R. J. (1999). Production of 2,3-butanediol by newly isolated *Enterobacter cloacae*. Appl. Microbiol. Biotechnol..

[cit41] Liakou V., Pateraki C., Palaiogeorgou A. M., Kopsahelis N., Machado de Castro A., Guimarães Freire D. M., Nychas G. J. E., Papanikolaou S., Koutinas A. (2018). Valorisation of fruit and vegetable waste from open markets for the production of 2,3-butanediol. Food Bioprod. Process..

[cit42] Lee S. J., Lee J. H., Yang X., Kim S. B., Lee J. H., Yoo H. Y., Park C., Kim S. W. (2015). Phenolic compounds: Strong inhibitors derived from lignocellulosic hydrolysate for 2,3-butanediol production by *Enterobacter aerogenes*. Biotechnol. J..

[cit43] Joo J., Lee S. J., Yoo H. Y., Kim Y., Jang M., Lee J., Han S. O., Kim S. W., Park C. (2016). Improved fermentation of lignocellulosic hydrolysates to 2,3-butanediol through investigation of effects of inhibitory compounds by *Enterobacter aerogenes*. Chem. Eng. J..

[cit44] Li F., Pan S., Yang L., Liao S., Yang D., Wang Q., Huang S. (2024). Efficient production 2,3-butanediol from biomass-derived sugars by *Raoultella ornithinolytica* TH-21, a newly isolated lignocellulose-degrading bacterium. Ind. Crops Prod..

[cit45] Li L., Li K., Wang Y., Chen C., Xu Y., Zhang L., Han B., Gao C., Tao F., Ma C., Xu P. (2015). Metabolic engineering of *Enterobacter cloacae* for high-yield production of enantiopure (2R,3R)-2,3-butanediol from lignocellulose-derived sugars. Metab. Eng..

[cit46] Ling H. Z., Cheng K. K., Ge J. P., Ping W. X. (2017). Corncob Mild Alkaline Pretreatment for High 2,3-Butanediol Production by Spent Liquor Recycle Process. Bioenergy Res..

[cit47] Guimarães P. M. R., François J., Parrou J. L., Teixeira J. A., Domingues L. (2008). Adaptive evolution of a lactose-consuming *Saccharomyces cerevisiae* recombinant. Appl. Environ. Microbiol..

[cit48] Wouter Wisselink H., Toirkens M. J., Wu Q., Pronk J. T., Van Maris A. J. A. (2009). Novel evolutionary engineering approach for accelerated utilization of glucose, xylose, and arabinose mixtures by engineered *Saccharomyces cerevisiae* strains. Appl. Environ. Microbiol..

[cit49] Sunwoo I. Y., Sukwong P., Jeong D. Y., Kim S. R., Jeong G. T., Kim S. K. (2019). Enhancement of galactose consumption rate in *Saccharomyces cerevisiae* CEN.PK2-1 by CRISPR Cas9 and adaptive evolution for fermentation of <i>Kappaphycus alvarezii hydrolysate. J. Biotechnol..

[cit50] Wang X. X., Hu H. Y., Liu D. H., Song Y. Q. (2016). The implementation of high fermentative 2,3-butanediol production from xylose by simultaneous additions of yeast extract, Na2EDTA, and acetic acid. N Biotechnol..

[cit51] Zhang C., Li W., Wang D., Guo X., Ma L., Xiao D. (2016). Production of 2,3-butanediol by *Enterobacter cloacae* from corncob-derived xylose. Turk. J. Biol..

[cit52] Guo X. W., Zhang Y., Li L. L., Guan X. Y., Guo J., Wu D. G., Chen Y. F., Xiao D. G. (2018). Improved xylose tolerance and 2,3-butanediol production of *Klebsiella pneumoniae* by directed evolution of rpoD and the mechanisms revealed by transcriptomics. Biotechnol. Biofuels.

[cit53] Kuenz A., Jäger M., Niemi H., Kallioinen M., Mänttäri M., Prüße U. (2020). Conversion of Xylose from Birch Hemicellulose Hydrolysate to 2,3-Butanediol with *Bacillus vallismortis*. Fermentation.

[cit54] Li L., Zhang L., Li K., Wang Y., Gao C., Han B., Ma C., Xu P. (2013). A newly isolated *Bacillus licheniformis* strain thermophilically produces 2,3-butanediol, a platform and fuel bio-chemical. Biotechnol. Biofuels.

[cit55] Jurchescu I.-M., Hamann J., Zhou X., Ortmann T., Kuenz A., Prüße U., Lang S. (2013). Enhanced 2,3-butanediol production in fed-batch cultures of free and immobilized *Bacillus licheniformis DSM 8785*. Appl. Microbiol. Biotechnol..

[cit56] Silva M. d. O., de Castro R. J. S. (2025). First-Order Degradation Kinetics of Phenolic Compounds and Antioxidant Properties of Fresh and Enzymatically Hydrolyzed Seriguela Pulp (Spondias purpurea L.). ACS Food Sci. Technol..

[cit57] Köntös Z. (2026). Kinetics of uncatalyzed hydrogen peroxide decomposition: Determination of rate constants, Arrhenius parameters. J. Chem. Res..

