# Supplementary material for: Enhanced arabinose utilization by adaptively evolved Klebsiella pneumoniae enables efficient 2,3-butanediol production from sugar beet pulp
Source: Sustain Energy Fuels. 2026 Jul 12;10(15):3715–32. doi: 10.1039/d6se00474a (PMC13356880; doi:10.1039/d6se00474a)

Supplementary Information (SI) for Sustainable Energy & Fuels.
This journal is © The Royal Society of Chemistry 2026

Enhanced arabinose utilization by adaptively evolved *Klebsiella pneumoniae* enables efficient 2,3-butanediol production from sugar beet pulp

Supplementary data

Supplementary Table 1: Comparison of BDO production by various evolved strains of *K. pneumoniae*

K. pneumoniae strain	Maximum cell OD_{600}	BDO		
		Titer (g/L)	Yield (g/g)	Productivity (g/L. h)
Parent strain	12.20	34.8	0.36	0.435
A1	12.34	35.9	0.37	0.449
A2	14.56	38.0	0.39	0.475
A3	13.70	41.3	0.42	0.516
A4	13.36	43.8	0.43	0.547

Supplementary Table 2: Comparison of cell OD (600 nm) and BDO production by *K. pneumoniae* A4 strain during fed-batch culture on pure sugar mixture and sugar rich hydrolysate.

Feedstock	Maximum cell OD_{600}	BDO		
		Titer (g/L)	Yield (g/g)	Productivity (g/L. h)
Arabinose rich mixture	36.2	84.9	0.39	0.88
Glucose rich mixture	38.1	96.7	0.39	0.94
Arabinose rich hydrolysate (non - pasteurized)	-	82.2	0.38	0.63
Arabinose rich hydrolysate (pasteurized)	-	79.21	0.38	0.78
Glucose rich hydrolysate (non - pasteurized)	-	92.4	0.38	0.58
Glucose rich	-	84.00	0.39	0.74

hydrolysate (pasteurized)				

Supplementary Figure 1: Time course profile of parent and ALE strains at 100 g/L arabinose showing arabinose consumption, OD_{600}, pH, and BDO formation. (A) parent strain, (B) A1 strain, (C) A2 strain, (D) A3 strain. Symbols: arabinose (▼), BDO (◆), OD600 (◇), pH (☆).

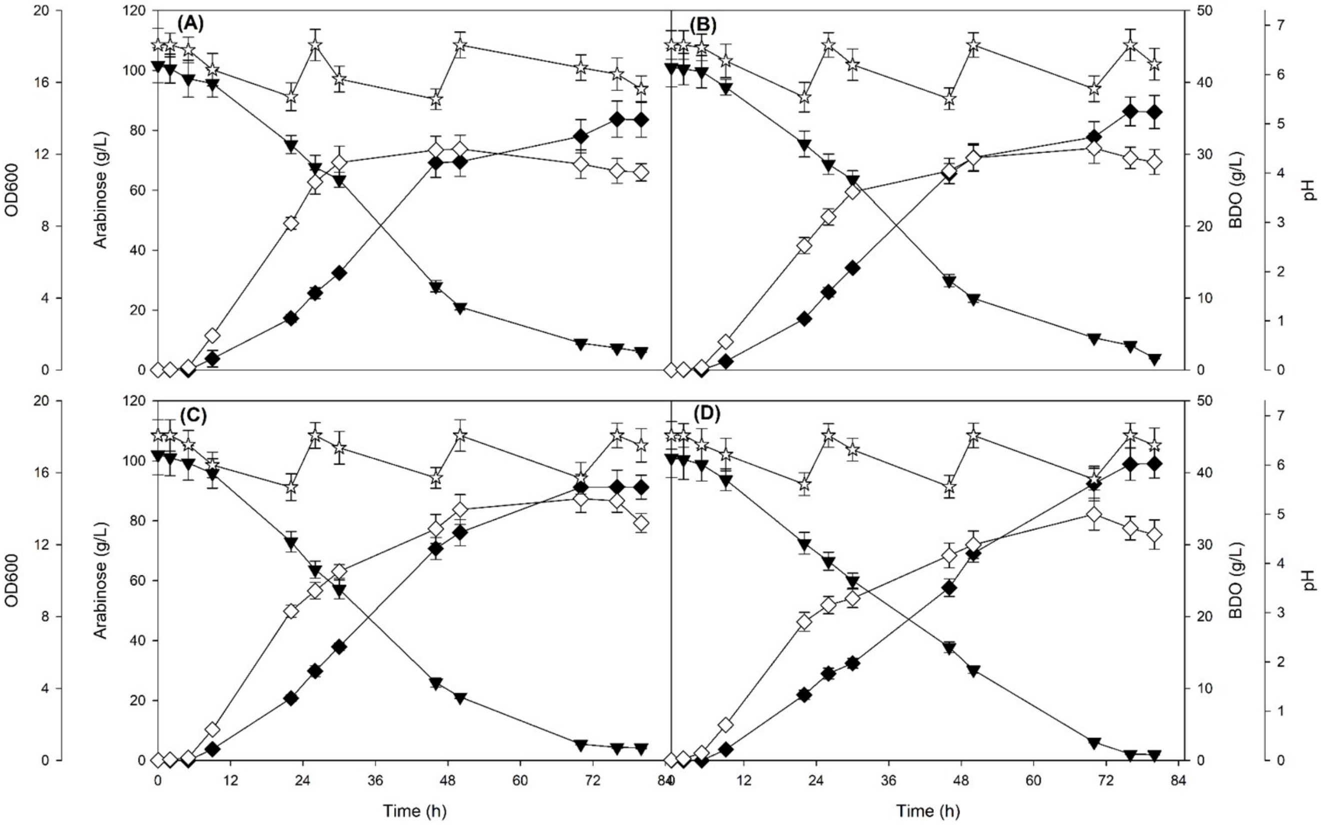

Supplementary Fig.2: Byproduct profile of parent and ALE strains at 100 g/L arabinose – succinic, lactic, formic, acetic acid, and ethanol. (A) parent strain, (B) A1 strain, (C) A2 strain, (D) A3 strain. Symbols: acetic acid (△), formic acid (⬢), succinic acid (▲), LA (⬡), ethanol (▽).

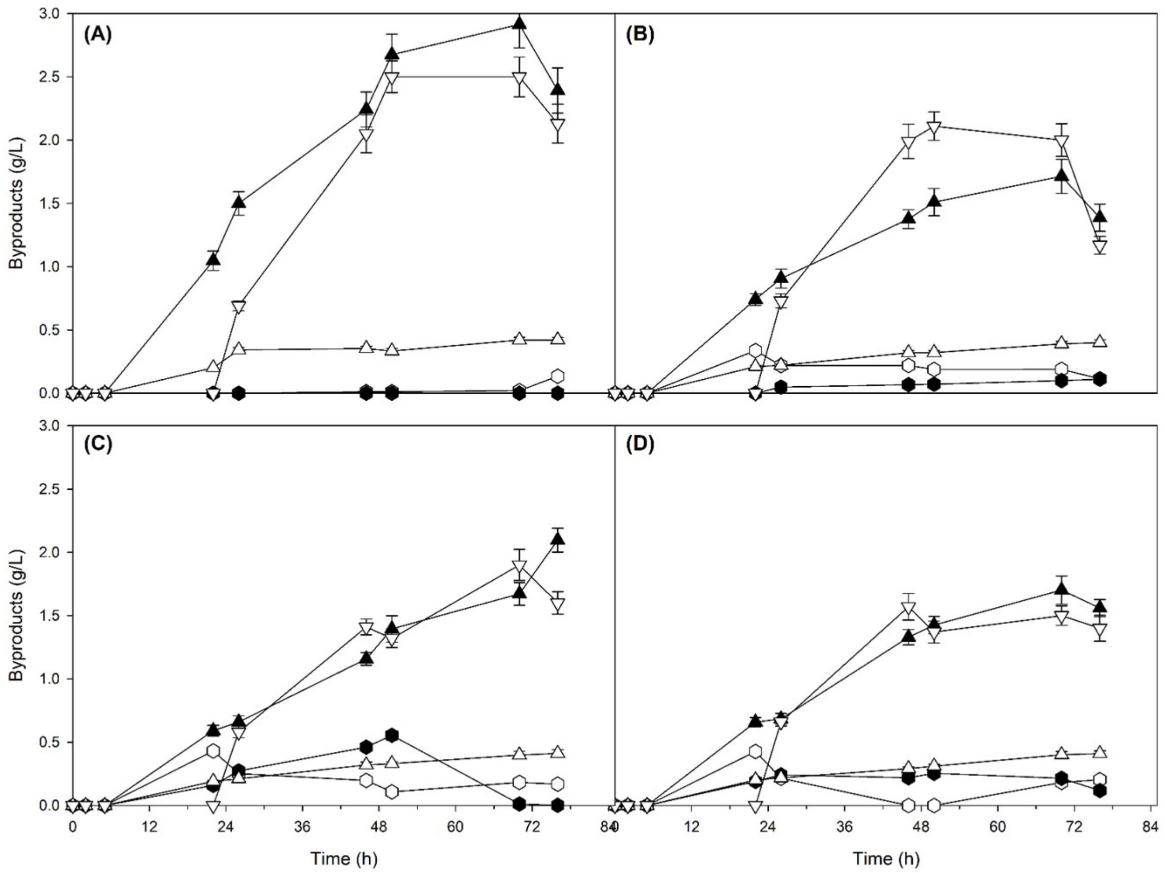

Supplement: SE-010-D6SE00474A-s001 [file SE-010-D6SE00474A-s001.pdf]
